# WGCNA combined with machine learning algorithms for analyzing key genes and immune cell infiltration in heart failure due to ischemic cardiomyopathy

**DOI:** 10.3389/fcvm.2023.1058834

**Published:** 2023-03-17

**Authors:** XiangJin Kong, HouRong Sun, KaiMing Wei, LingWei Meng, Xin Lv, ChuanZhen Liu, FuShun Lin, XingHua Gu

**Affiliations:** ^1^ Qilu Hospital, Cheeloo College of Medicine, Shandong University, Jinan, China; ^2^ Department of Cardiovascular Surgery, Qilu Hospital of Shandong University, Jinan, China

**Keywords:** ischemic cardiomyopathy (ICM), heart failure (HF), machine learning (ML), immune cell infiltration, weighted gene co-expression network analysis (WGCNA)

## Abstract

**Background:**

Ischemic cardiomyopathy (ICM) induced heart failure (HF) is one of the most common causes of death worldwide. This study aimed to find candidate genes for ICM-HF and to identify relevant biomarkers by machine learning (ML).

**Methods:**

The expression data of ICM-HF and normal samples were downloaded from Gene Expression Omnibus (GEO) database. Differentially expressed genes (DEGs) between ICM-HF and normal group were identified. Kyoto Encyclopedia of Genes and Genomes (KEGG) pathway enrichment and gene ontology (GO) annotation analysis, protein–protein interaction (PPI) network, gene pathway enrichment analysis (GSEA), and single-sample gene set enrichment analysis (ssGSEA) were performed. Weighted gene co-expression network analysis (WGCNA) was applied to screen for disease-associated modules, and relevant genes were derived using four ML algorithms. The diagnostic values of candidate genes were assessed using receiver operating characteristic (ROC) curves. The immune cell infiltration analysis was performed between the ICM-HF and normal group. Validation was performed using another gene set.

**Results:**

A total of 313 DEGs were identified between ICM-HF and normal group of GSE57345, which were mainly enriched in biological processes and pathways related to cell cycle regulation, lipid metabolism pathways, immune response pathways, and intrinsic organelle damage regulation. GSEA results showed positive correlations with pathways such as cholesterol metabolism in the ICM-HF group compared to normal group and lipid metabolism in adipocytes. GSEA results also showed a positive correlation with pathways such as cholesterol metabolism and a negative correlation with pathways such as lipolytic presentation in adipocytes compared to normal group. Combining multiple ML and cytohubba algorithms yielded 11 relevant genes. After validation using the GSE42955 validation sets, the 7 genes obtained by the machine learning algorithm were well verified. The immune cell infiltration analysis showed significant differences in mast cells, plasma cells, naive B cells, and NK cells.

**Conclusion:**

Combined analysis using WGCNA and ML identified coiled-coil-helix-coiled-coil-helix domain containing 4 (CHCHD4), transmembrane protein 53 (TMEM53), acid phosphatase 3 (ACPP), aminoadipate-semialdehyde dehydrogenase (AASDH), purinergic receptor P2Y1 (P2RY1), caspase 3 (CASP3) and aquaporin 7 (AQP7) as potential biomarkers of ICM-HF. ICM-HF may be closely related to pathways such as mitochondrial damage and disorders of lipid metabolism, while the infiltration of multiple immune cells was identified to play a critical role in the progression of the disease.

## 1. Introduction

Heart failure (HF) is a complex clinical syndrome and the end-stage manifestation of cardiovascular disease (1, 2). Ischemic heart disease refers to myocardial degeneration, necrosis and fibrosis caused by coronary artery disease, which leads to severe left ventricular dysfunction (LVEF ≤ 35%–40%) (3). The alterations in neurohumoral, cellular, and molecular mechanisms are triggered by the structural damage and decompensation of the heart and act as a network to maintain its original normal physiological functions. These coordinated, complex processes lead to excessive volume overload, increased sympathetic activity, and circulatory redistribution and result in the distinct, parallel development of clinical signs and symptoms (4). Depending on the cause, HF is divided into ischemic HF caused by ischemic cardiomyopathy (ICM) and non-ischemic HF (5). ICM refers to the damage to the heart muscle caused by ischemia, where the heart is unable to pump blood properly. According to the WHO, ICM is the leading cause of death worldwide (6). Despite new drugs and surgical advances in the treatment of ICM, the prognosis for ischemic HF caused by coronary artery disease remains poor, with a five-year mortality rate of 40%–50% (7). A recent report from China showed that the prevalence of HF among residents aged ≥35 years was 1.3% (8). Thus, research targeting HF, especially ischemic HF, is of great importance. With the advancements in science and technology, we have developed a new understanding of HF caused by ICM, i.e., genetic alterations and immune environmental factors are jointly involved in the progression of the pathological process.

With the advancements in bioinformatics, the available microarray data can be used to identify hub genes, interaction networks, and pathways in ischemic HF. While traditional assays have certain limitations, weighted gene co-expression network analysis (WGCNA) is a highly systematic bioinformatics method (9). WGCNA may be applied to construct expression profiles of mRNAs in HF triggered by ICM by combining multiple informatics approaches to screen for modules and genes that are highly correlated with the disease to reveal potential molecular mechanisms. It can help provide new ideas for the diagnosis and treatment of the disease. WGCNA constructs scale-free networks by linking gene expression levels to clinical features and is commonly used for the bioanalysis of various systems. We first normalized the samples and then removed outlier samples to ensure reliable results in network construction. Soft threshold power had to be selected according to the standard scale-free network, and all differential genes were calculated using the power function. Machine learning (ML) method has very significant advantages in the processing of big data (10). Algorithms for ML analyze training data to uncover hidden patterns, build models, and then make predictions using the most accurate of these patterns. In fact, existing technology, such as support vector machine recursive feature elimination (SVM-RFE) and random forest (RF), have been applied to problems in genomics, proteomics, systems biology and other fields (11). ML methods are distinguished by their capacity to examine large amounts of data in order to discover correlations, provide explanations. These ML methods can assist in enhancing the dependability, performance, predictability and precision of diagnostic systems (12). Recent research suggests that the application of ML techniques may have the potential to improve heart failure outcomes and management by improving existing diagnostic and therapeutic support systems (13).

In the past decades, high-throughput platforms for analyzing gene expression, such as microarray technology, have been widely used to screen for genetic alterations at the genomic level, which helps us identify differentially expressed genes (DEGs), functions and pathways associated with disease pathogenesis and progression. We identified DEGs by using R (v4.0.1) software with Limma package (14) between ICM-HF myocardial tissue and normal tissue. WGCNA, gene ontology (GO), Kyoto gene and genome encyclopedia (KEGG) pathway enrichment analyses were performed and protein-protein interaction (PPI) networks were constructed and various ML approaches were used for further screening to explore the molecular mechanisms behind ICM-HF. Subsequently, we screened the most important modules of the PPI network built by DEG and the hub genes was screened by ML for further discussion. The aim of this study is to explore the underlying molecular mechanisms in ICM through a combination of several common analytical methods and ML approaches. Future research in the field of cardiovascular disease may benefit from the ideas and methods generated by our work.

## 2. Materials and methods

### 2.1. Data acquisition and preprocessing

**Figure 1:**
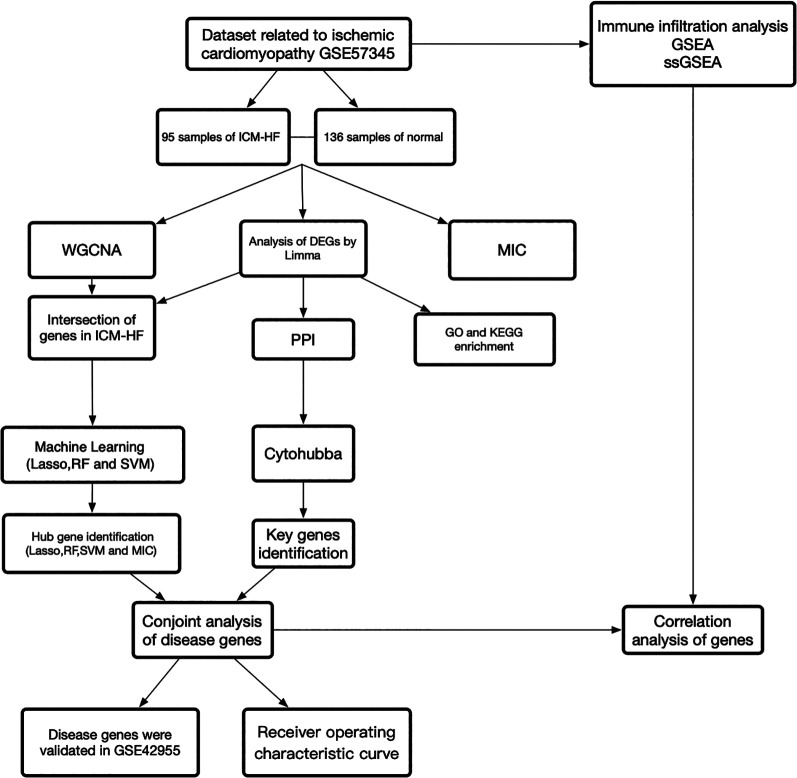
Study flowchart, Figure 1 is a summary of our study as a whole.

Figure 1 depicts the study flowchart. A sample of 136 normal samples and 95 samples of ICM-HF from the GSE57345 (15) dataset. The GSE42955 (16) dataset was downloaded from the GEO database (https://www.ncbi.nlm.nih.gov/geo/) for validation of the results. ICM-HF was determined by medical history and pathological examination of the explanted hearts (15). Information on the datasets was displayed in Supplementary Table S1. Batch effects were removed using R (v4.0.1). Gene annotation was completed based on GPL9052 Illumina Genome Analyzer (Homo sapiens) and GPL6244 Affymetrix Human Gene 1.0 ST Array (Homo sapiens). It should be noted that if a gene has multiple probe loci, the average value of the probe loci is used as the gene expression level when converting probe ID to gene symbol. On the basis of the annotation files from the respective platforms, probe IDs were converted to gene symbols and probes that did not correspond to gene symbols were removed.

### 2.2. Identification of differentially expressed genes

We compared ICM-HF subjects with normal using R (v4.0.1). We used the limma package in R to distinguish between differentially expressed genes (DEGs) and then set |log2 (fold change)| ≥ 0.5 and adjusted *p* < 0.05 as the threshold for DEGs, followed by WGCNA and identification of modules. We also used the MIC algorithm implemented in the minepy class library in Python to screen genes.

### 2.3. Protein–protein interaction analysis network construction and module analysis

We entered DEGs into the STRING database (http://string-db.org) to collect interactions of target proteins with a medium confidence score >0.4 and constructed a protein–protein interaction (PPI) network (v3.9.0) using Cytoscape software. In addition, we used the Cytoscape plug-in software “cytoHubba” to identify related genes based on mixed character calculations.

### 2.4. Functional enrichment analysis of DEGs

Gene Ontology (GO) and Kyoto Encyclopedia of Genes and Genomes (KEGG) pathway enrichment analysis are two very important components of bioinformatics analysis. It is difficult to describe the function and relationship among these genes only by gene names. This allows for better insight into the pathways behind the genes. Therefore, we performed a visual analysis in R to analyze all genes in the modules of interest and to identify possible mechanisms by which the module genes play a role in the clinical features of interest. Cutoff criteria were set at a *p*-value <0.05 and a false discovery rate (FDR) <0.1.

### 2.5. Machine learning analysis of disease genes

Gene fetching intersections using DEGs and WGCNA were used to select gene features using the minimum absolute shrinkage and selection operator (LASSO) algorithms of the glmnet R package (17) and the e1071 package (18), LASSO is a regression method for selecting a variable to improve the predictive accuracy and is also a regression technique for variable selection and regularization to improve the predictive accuracy and comprehensibility of a statistical model (19), respectively, and the support vector machine recursive feature elimination (SVM-RFE) method (20). Support vector machines (SVM) are a powerful tool to analyze data with a number of predictors approximately equal or larger than the number of observations (20). The “randomForest” R package (21) was used to perform the random forest (RF) analysis. RF is an appropriate approach with the benefits of no limits on variable conditions and better accuracy, sensitivity, and specificity, which can be used to predict continuous variables and provide forecasts without apparent variations (22). The use of Maximal Information Coefficient Maximum mutual information coefficient (MIC) to measure the degree of association between two genes, linear or nonlinear, is more accurate than Mutual Information (MI) mutual information. Next, the intersection-related genes were derived.

### 2.6. The receiver operating characteristic curve evaluation of candidate genes and tests of relative expression of genes

Receiver operating characteristic (ROC) curves were established to assess the diagnostic value of candidate genes and columnar maps for ICM-HF, and the area under the curve (AUC) and 95% confidence interval (CI) were calculated to quantify their value. AUC > 0.70 was considered the ideal diagnostic value. Differential expression in the experimental and validation groups was then assessed separately using a nonparametric test and visualized through R.

### 2.7. Immune infiltration analysis

CIBERSORT is a computational method for determining the proportion of immune cells in HF and controls using tissue gene expression profiles to identify different immune cell proportions (23). We performed immune cell infiltration analysis using the “Cibersort” R software package (23). Bar graphs were used to visualize the proportion of each immune cell type in different samples. A comparison of the proportion of different types of immune cells between HF and control groups was visualized by vioplot. Heatmaps depicting the correlation of 22 types of infiltrating immune cells were created using the “corrplot” R package (24).

### 2.8. GSEA analysis and ssGSEA analysis

For gene set enrichment analysis (GSEA), we obtained the GSEA software (version 3.0) from the GSEA website (DOI: 10.1073/pnas.0506580102, http://software.broadinstitute.org/gsea/index.jsp). We then divided the samples into two groups according to the occurrence of HF and then downloaded the GSEA software from the Molecular Signatures Database (DOI: 10.1093/bioinformatics/btr260, http://www.gsea-msigdb.org/gsea/downloads.jsp) downloaded the c2.cp.kegg.v7.4.symbols.gmt subset to evaluate relevant pathways and molecular mechanisms based on gene expression profiles and phenotypic groupings, setting a minimum gene set of 5 and a maximum gene set of 5,000, with a *p*-value of <0.05 (as needed) and an FDR of <0.25 (as needed) were considered statistically significant.

## 3. Results

### 3.1. Transcriptome profile analysis of the ICM samples and normal samples

**Figure 2:**
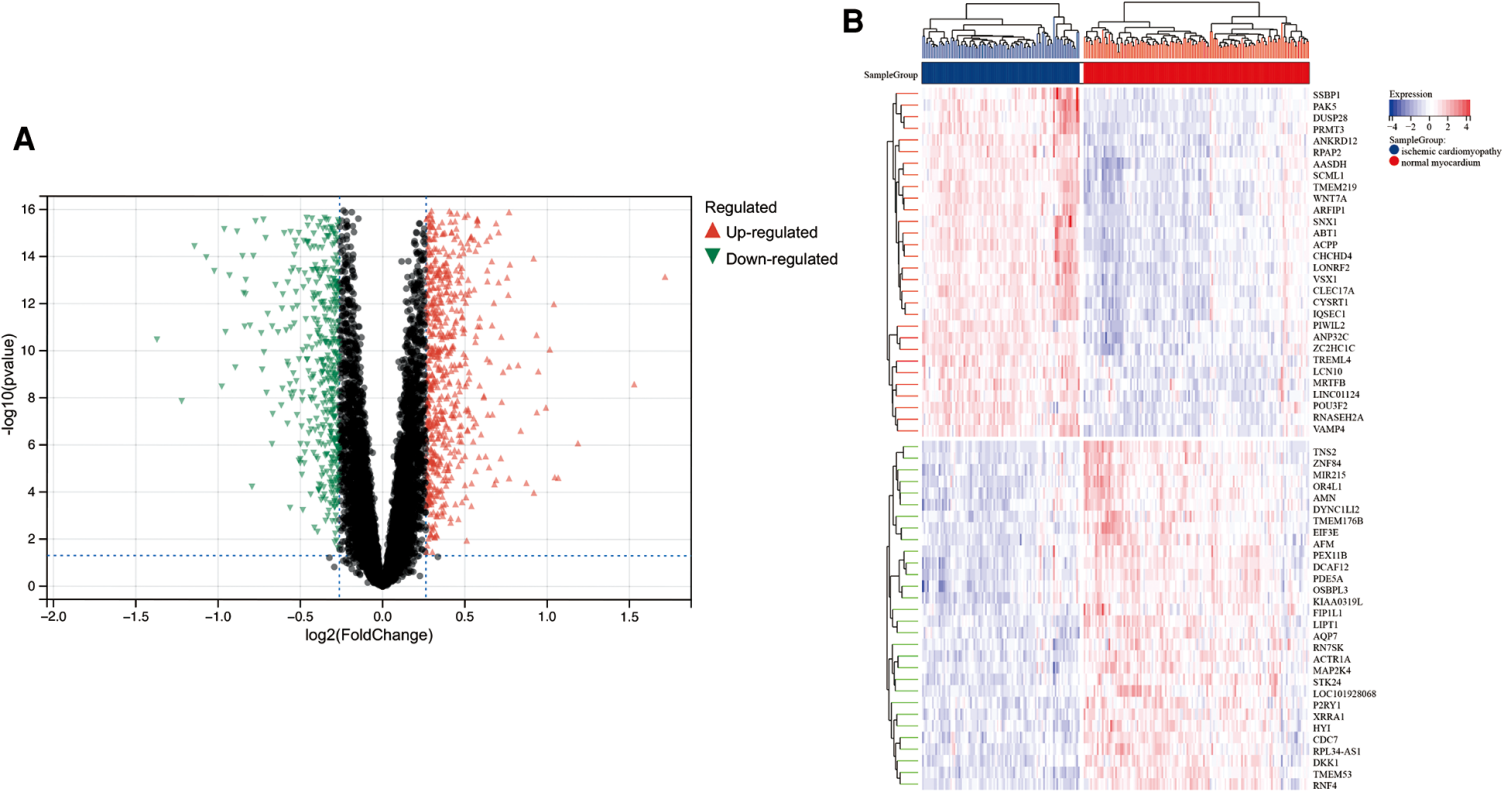
Limma analysis of ICM-HF and the normal group. (**A**) Volcano plot of differentially expressed genes (DEGs) in GSE57345, set |log2(FC)| ≥ 0.5. Red dots are upregulated genes, and green dots are downregulated genes. (**B**) A heatmap showing the top 30 upregulated and the top 30 downregulated genes in ICM-HF and normal groups.

**Figure 3:**
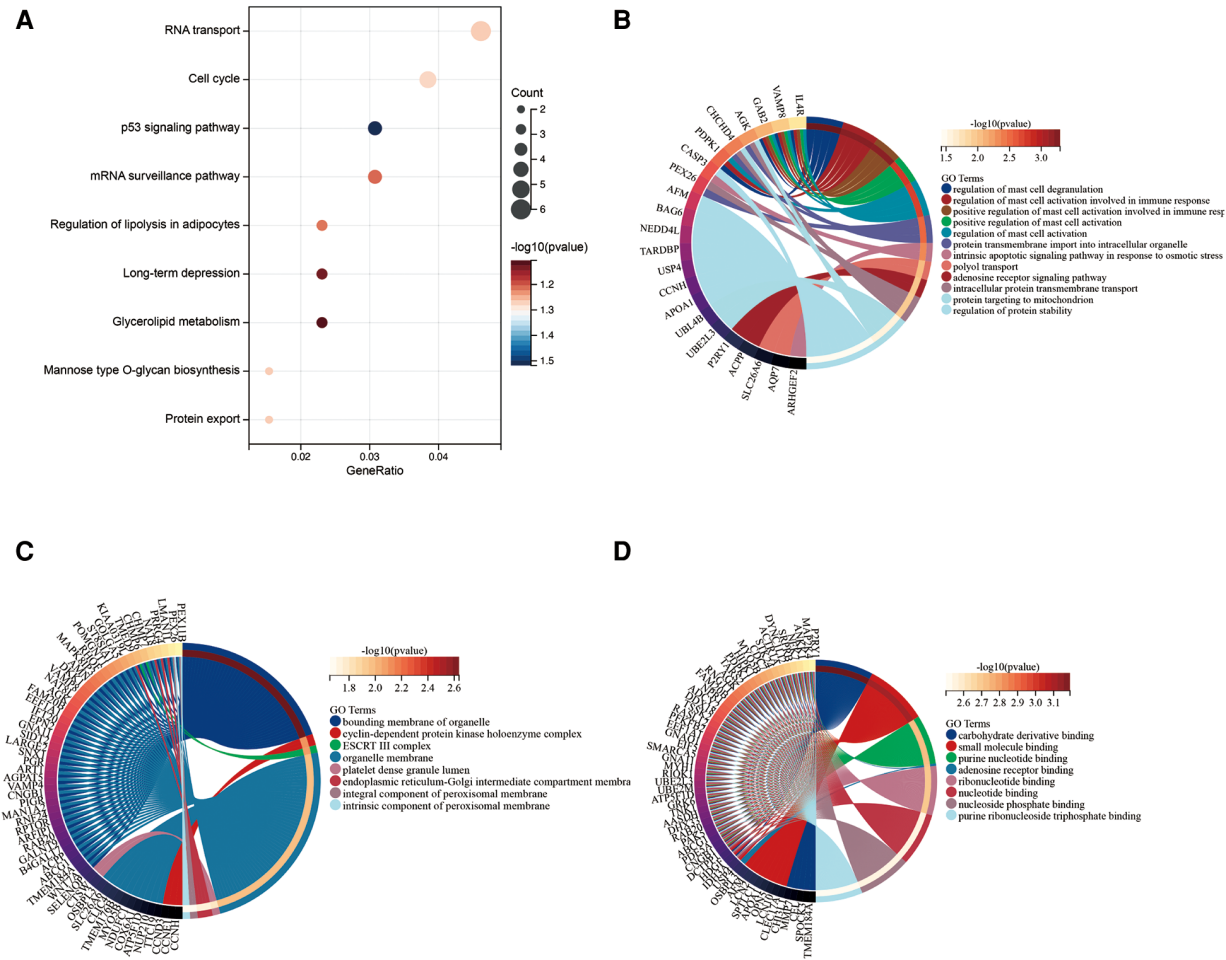
Functional enrichment of genes in the object module. The *x*-axis shows the number of ratios of genes, and the *y*-axis shows the pathway terms. The −log10 (*p*-value) of each term is colored according to the legend. (**A**) Kyoto encyclopedia of genes and genomes (KEGG) pathway analysis. (**B–D**) Gene ontology (GO) analysis.

A total of 313 DEGs were identified in GSE57345, and 184 upregulated and 129 downregulated genes were identified in the ICM-HF group (Figure 2A). The heatmap shows the expression profiles of the top 30 upregulated DEGs and the top 30 downregulated DEGs (Figure 2B). We performed GO and KEGG pathway analysis to investigate the biological function of DEGs. Our KEGG analysis revealed that differential genes were mainly enriched in the p53 signaling pathway, cell cycle regulation, and lipid metabolism pathway (Figure 3A). BP analysis revealed that differential genes were mainly enriched in numerous immune response pathways, intrinsic organelle damage regulation, and protein transport (Figure 3B). CC analysis revealed that differential genes were enriched in numerous organelle peroxidase and organelle membrane regulation (Figure 3C). MF enrichment the analysis showed that the differential genes were enriched in the metabolism of nucleotides (Figure 3D).

### 3.2. Weighted gene co-expression network analysis screens for key modules

**Figure 4:**
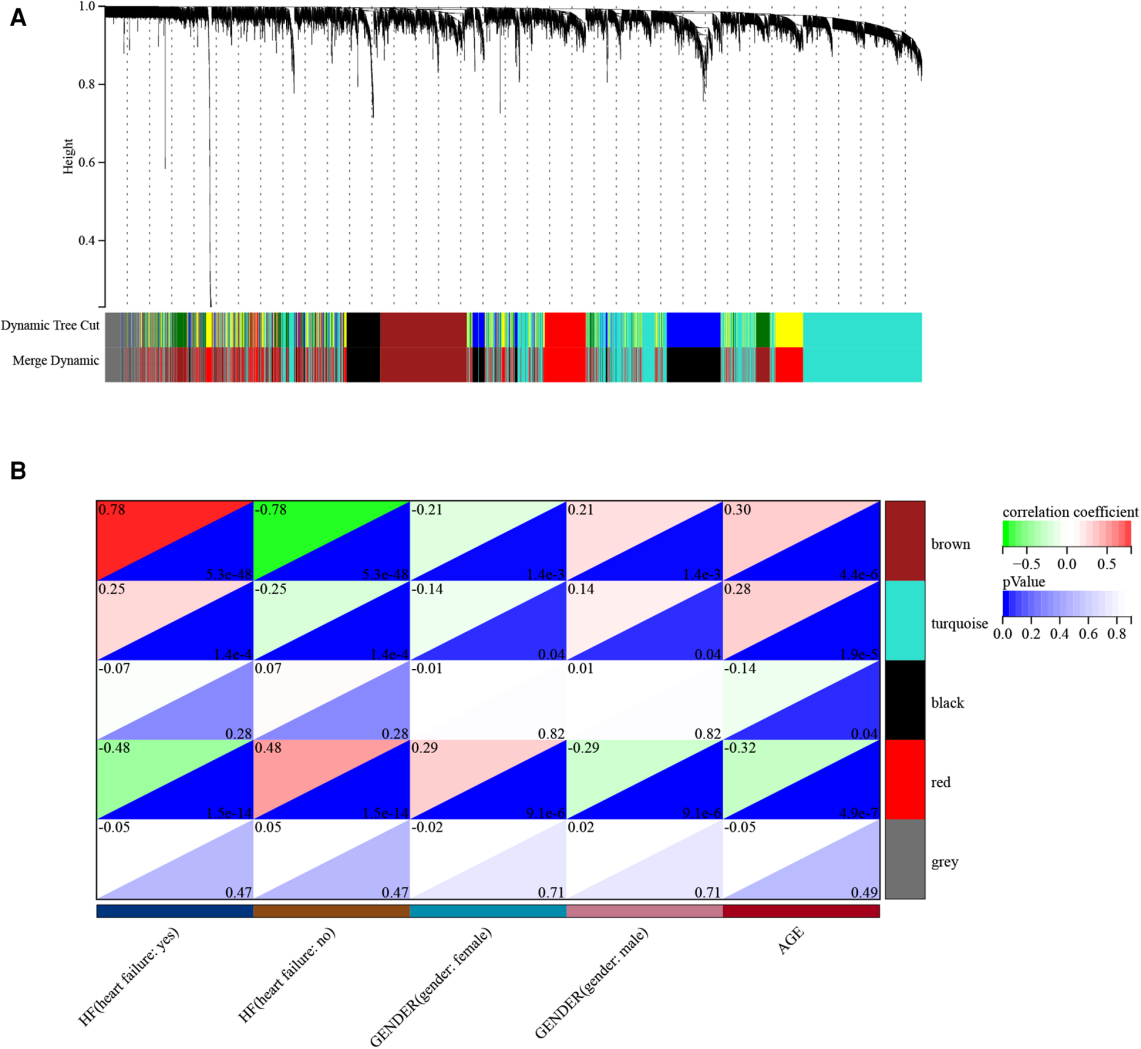
Demonstration of the WGCNA process. (**A**) Gene and trait clustering dendrograms. Gene clustering trees (dendrograms) obtained by hierarchical clustering of neighbor-based differences. (**B**) Module feature association. Each row corresponds to a module feature, and each column corresponds to a clinical feature. Each cell contains the corresponding correlation in the first row and the corresponding *p*-value in the second row. The table is color-coded by correlation according to the color legend.

Before constructing the weighted co-expression network, we selected the soft threshold β parameter as the appropriate weighting parameter for the neighbor-joining function. After calculation, we set the soft threshold β to 6 and chose a correlation coefficient close to 0.86 to construct the gene modules (Supplementary Figure S1A,B). In total, about five gene modules were identified using dynamic tree cutting in all samples (Figure 4A). The sensitivity was set to 3. In addition, we merged modules with a distance of less than 0.5, resulting in five co-expression modules; notably, the gray module was regarded as the set of genes that could not be assigned to any module and the brown module was considered the most significant gene module (Figure 4B). A total of 1,288 genes were identified in the brown gene module. Brown module membership and gene significance were significantly positively correlated (*r*^2^ = 0.82, *p* = 9.9E−324) (Supplementary Figure S1E).

### 3.3. Establishment of PPI protein interactions network to screen out key HUB genes

**Figure 5:**
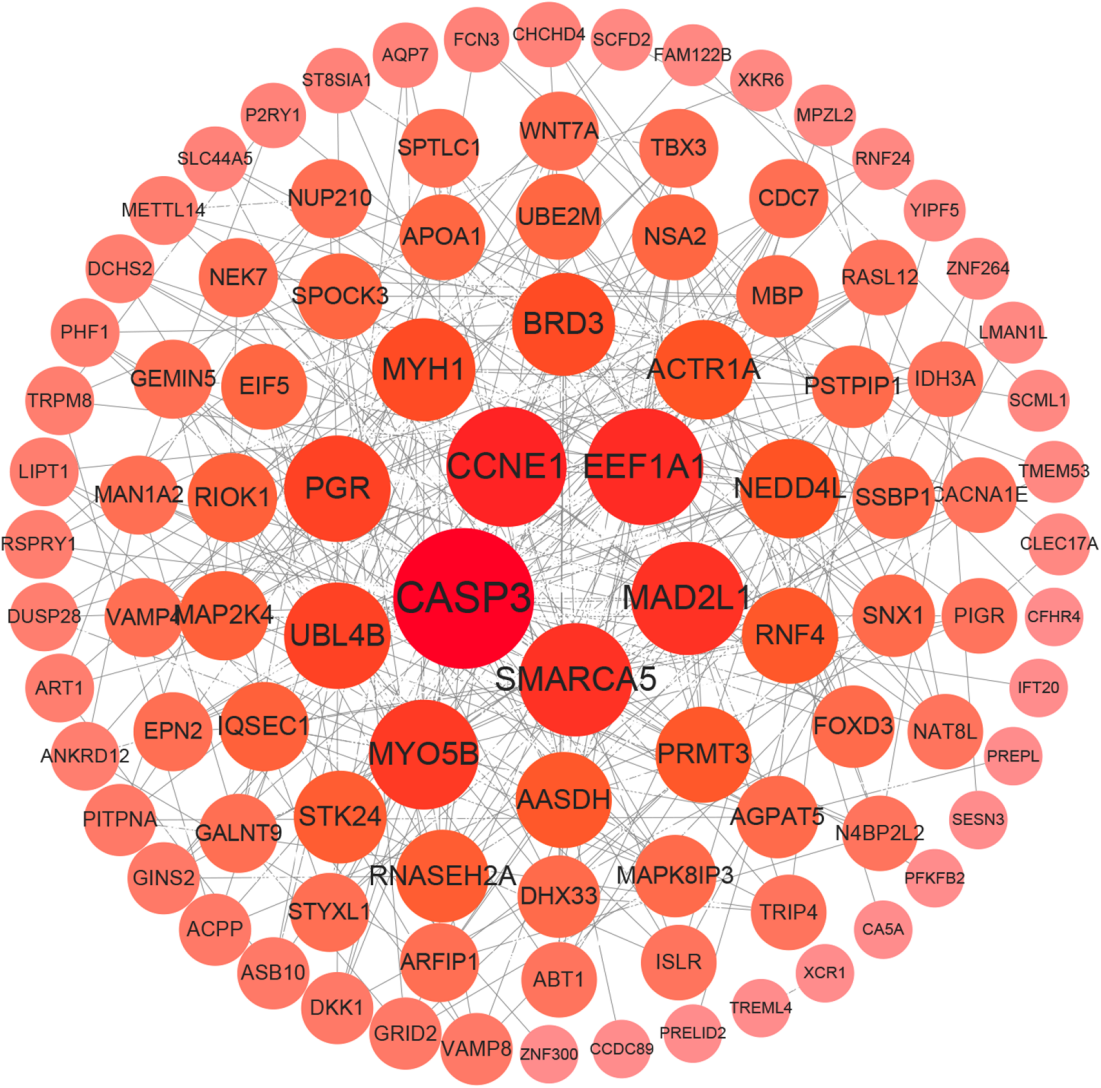
Protein–protein interaction (PPI) analysis. PPI network of DEGs. The edges represent the interactions between two genes. Degrees are used to describe the importance of protein nodes in the network; deep red colors indicate high degrees, whereas light red colors indicate low degrees.

In order to obtain a protein interaction network map between differential genes, the 313 genes from the limma analysis were entered into the STRING database and the PPI (https://cn.string-db.org) network was obtained. The PPI network (v3.9.0) was constructed using Cytoscape software (Figure 5), in order to further screen for key hub genes, the *Stress*, *MCC*, *Degree*, *EPC*, *EcCentricity*, *Radiality*, *Closeness* and *Betweenness* algorithms were used to calculate the associated gene scores (Supplementary Figure S2A–G). The UpSet graph was used to filter out five common HF-associated genes (Supplementary Figure S3). They are G protein subunit alpha O1 (GNAO1), cyclin H (CCNH), caspase 3 (CASP3), mitotic arrest deficient 2 like 1 (MAD2L1) and cyclin E1 (CCNE1).

### 3.4. Identification of key disease genes by machine learning

**Figure 6:**
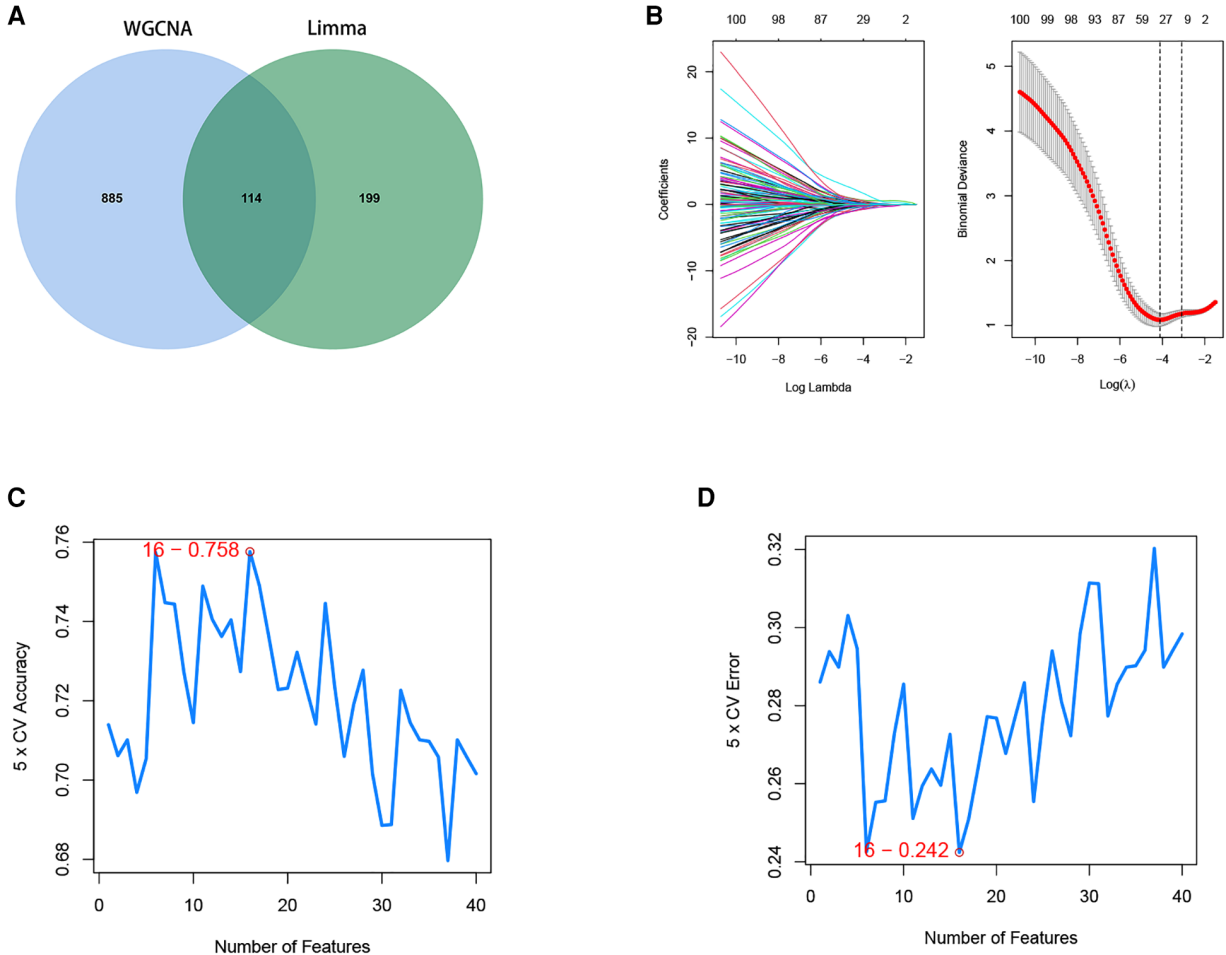
Screening of disease intersection genes and machine learning algorithm to identify the optimal feature genes. (**A**) Strong correlation module of WGCNA and Limma analysis of differential genes to do a Venn diagram screening of intersecting genes. (**B**) LASSO coefficient profiles of the candidate optimal feature genes and the optimal lambda was determined when the partial likelihood deviance reached the minimum value. Each coefficient curve in the left picture represents a single gene. The solid vertical lines in right picture represent the partial likelihood deviance, and the number of genes (*n* = 30) corresponding to the lowest point of the cure is the most suitable for LASSO. (**C,D**) The SVM-RFE algorithm was used to further candidate optimal feature genes with the highest accuracy and lowest error obtained in the curves. The *x*-axis shows the number of feature selections, and the *y*-axis shows the prediction accuracy. Sixteen gene features were identified through SVM-RFE analysis with an accuracy of 0.758 and an error of 0.242.

**Figure 7:**
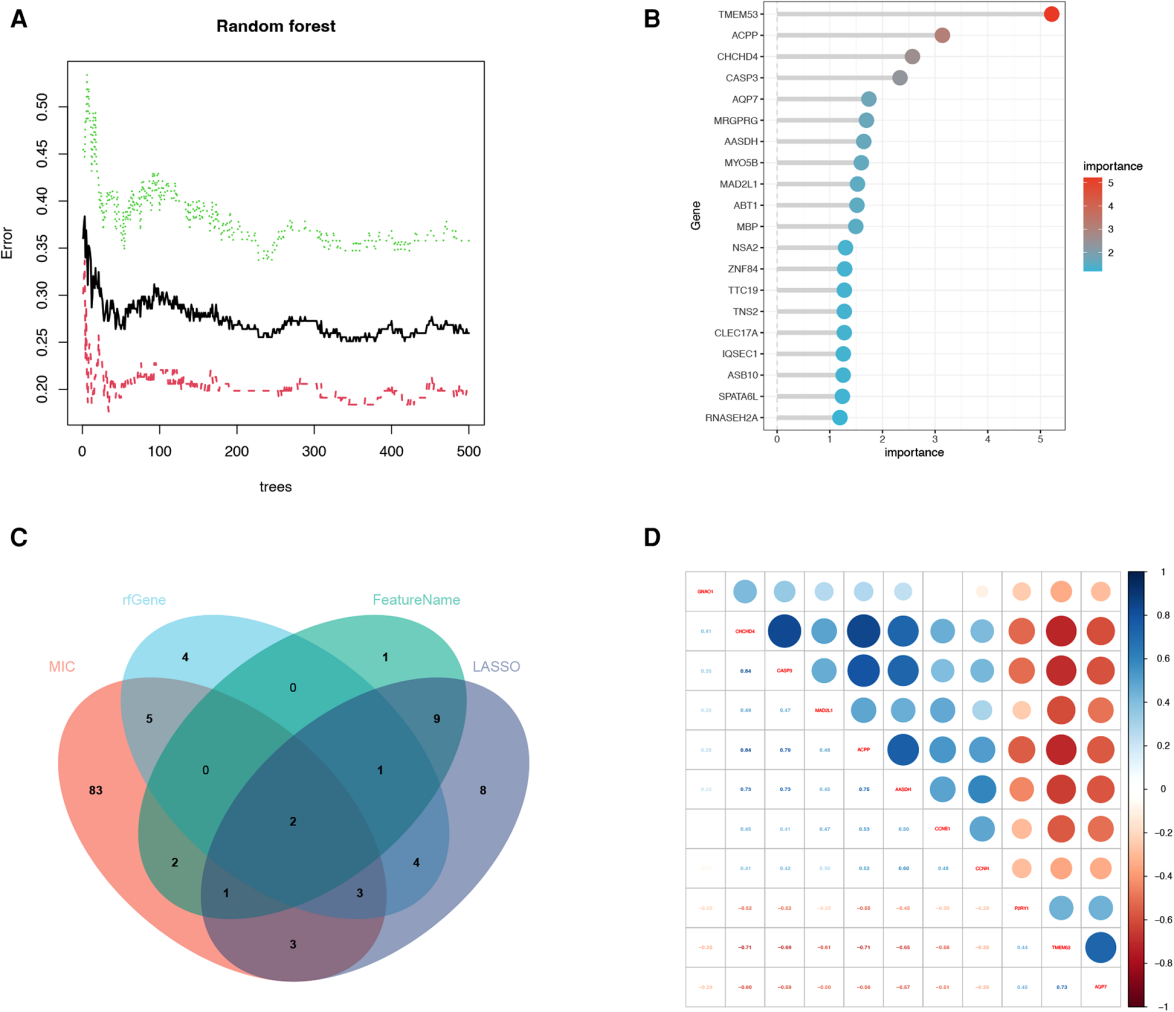
Machine learning algorithm and correlation heatmap. (**A**) Relative importance of overlapping candidate genes calculated in random forest (Top 10 genes’ importance >2). Importance indexes on the *x*-axis and genetic variables are plotted on the *y*-axis. (**B**) Relative importance of overlapping candidate genes calculated in random forest. Importance indexes on the *x*-axis and genetic variables are plotted on the *y*-axis. (**C**) Four machine learning algorithm genes screened for intersection genes using Venn diagram. (**D**) The correlation heatmap of machine learning algorithm-related genes and cytohubba algorithm-related genes. Red dots represent negative correlation between two genes, blue represents positive correlation between two genes. The absolute value corresponding to the dot is the correlation coefficient of the two genes.

**Figure 8:**
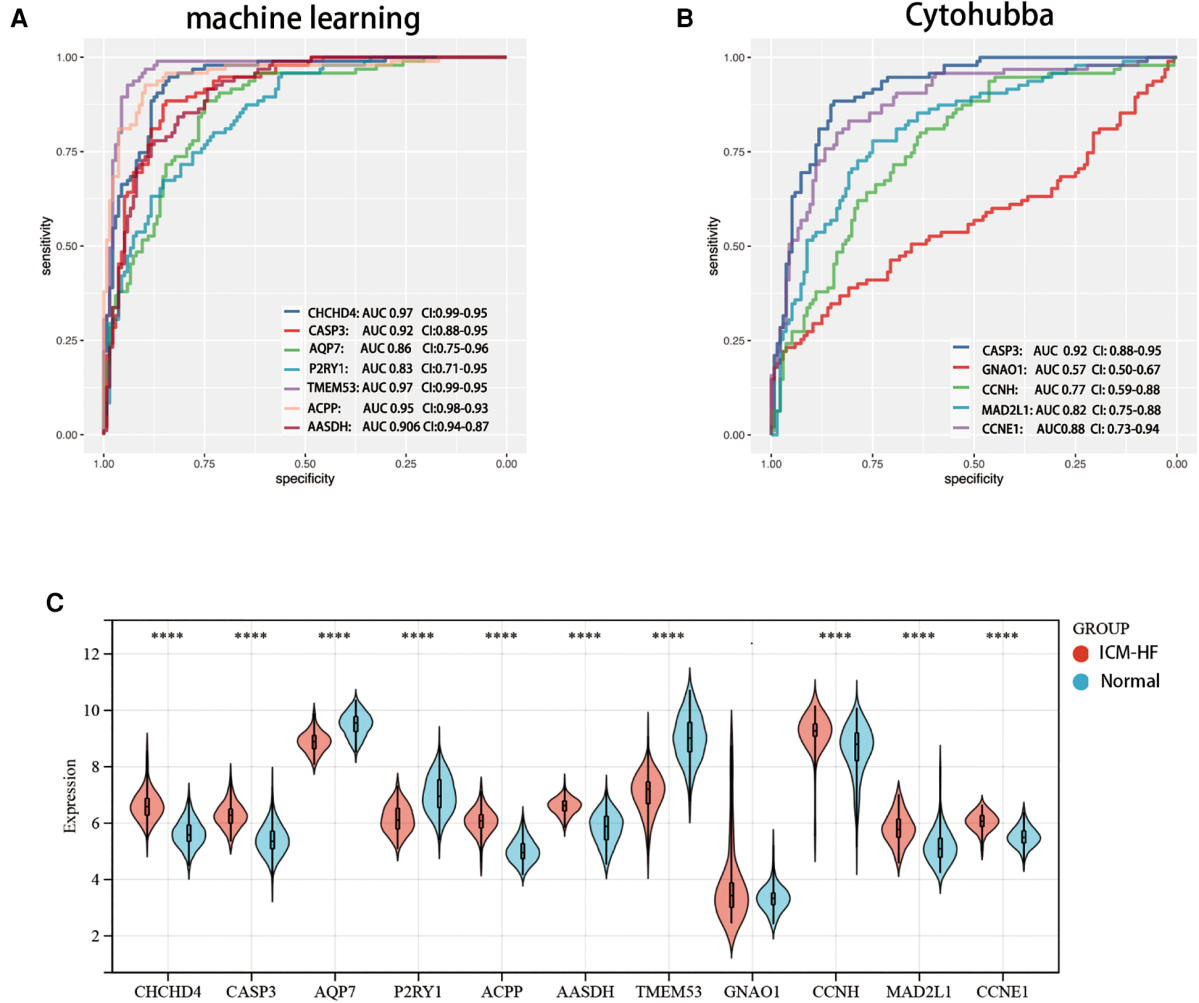
Verification of expression and diagnostic efficacy in predicting ICM-HF progression of optimal feature genes. (**A**) Validation of ROC of genes screened by a machine learning algorithm. (**B**) ROC validation of genes related to the cytohubba algorithm. (**C**) Violin plot of the expression of the relevant genes in the experimental set GSE57345. *p < 0.05, **p < 0.01, ***p < 0.001, Wilcoxon rank-sum test.

A total of 114 disease genes were obtained by taking the common genes of DEGs and WGCNA (Figure 6A). For further training of the above mentioned genes, these 114 genes were input into LASSO, RF algorithm and SVM-RFE algorithm were performed on GSE57345. A total of 31 genes were derived from the LASSO algorithm (Figure 6B). The top 16 genes from the SVM-RFE calculation were the most significant, with an accuracy of 0.758 (Figure 6C) and an error incidence of 0.242 (Figure 6D). The RF algorithm yielded 133 genes, and the top 20 in importance were taken as the resultant genes (Figures 7A,B). The three algorithms were then intersected with the ML MIC algorithm for a Venn diagram, and the genes with the intersection of the four algorithms were taken as the key disease genes (Figure 8C), yielding a total of seven genes. They are coiled-coil-helix-coiled-coil helix domain containing 4 (CHCHD4), transmembrane protein 53 (TMEM53), acid phosphatase 3 (ACPP), aminoadipate-semialdehyde dehydrogenase (AASDH), purinergic receptor P2Y1 (P2RY1), caspase 3 (CASP3), aquaporin 7 (AQP7). Among them, CHCHD4 and CASP3 genes are the causative genes common to all ML algorithms, with CASP3 being the gene shared by multiple cytohubba algorithm-related genes. We evaluated the correlation between the genes derived from cytohubba and ML algorithms (Figure 8D). Red dots represent negative correlation between two genes, blue represents positive correlation between two genes. The absolute value corresponding to the dot is the correlation coefficient of the two genes.

### 3.5. Establishment of ROC to assess the reliability of candidate genes and the relative expression of disease and experimental groups

We further evaluated the diagnostic values of TMEM53, ACPP, AASDH, P2RY1, CASP3, AQP7, GNAO1, CCNH, MAD2L1 and CCNE1 in GSE57345 using ROC curves, in order to improve their diagnostic performance, so we chose AUC > 0.7 as inclusion criteria. Demonstrating that these optimal feature genes have a high diagnostic value for ICM-HF and permit the estimation of progression (Figures 8A,B). The relative expression of genes in the experimental cohort was observed using a nonparametric test (Figure 8C) and validated using a validation set, the expression of GNAO1 was not statistically significant between the two groups (Figure 8C). CHCHD4, CASP3, ACPP, AASDH, CCNH, MAD2L1, and CCNE1 were all significantly upregulated in the ICM-HF group, and TMEM53, P2RY1, and AQP7 were significantly downregulated in the ICM-HF group.

**Figure 9:**
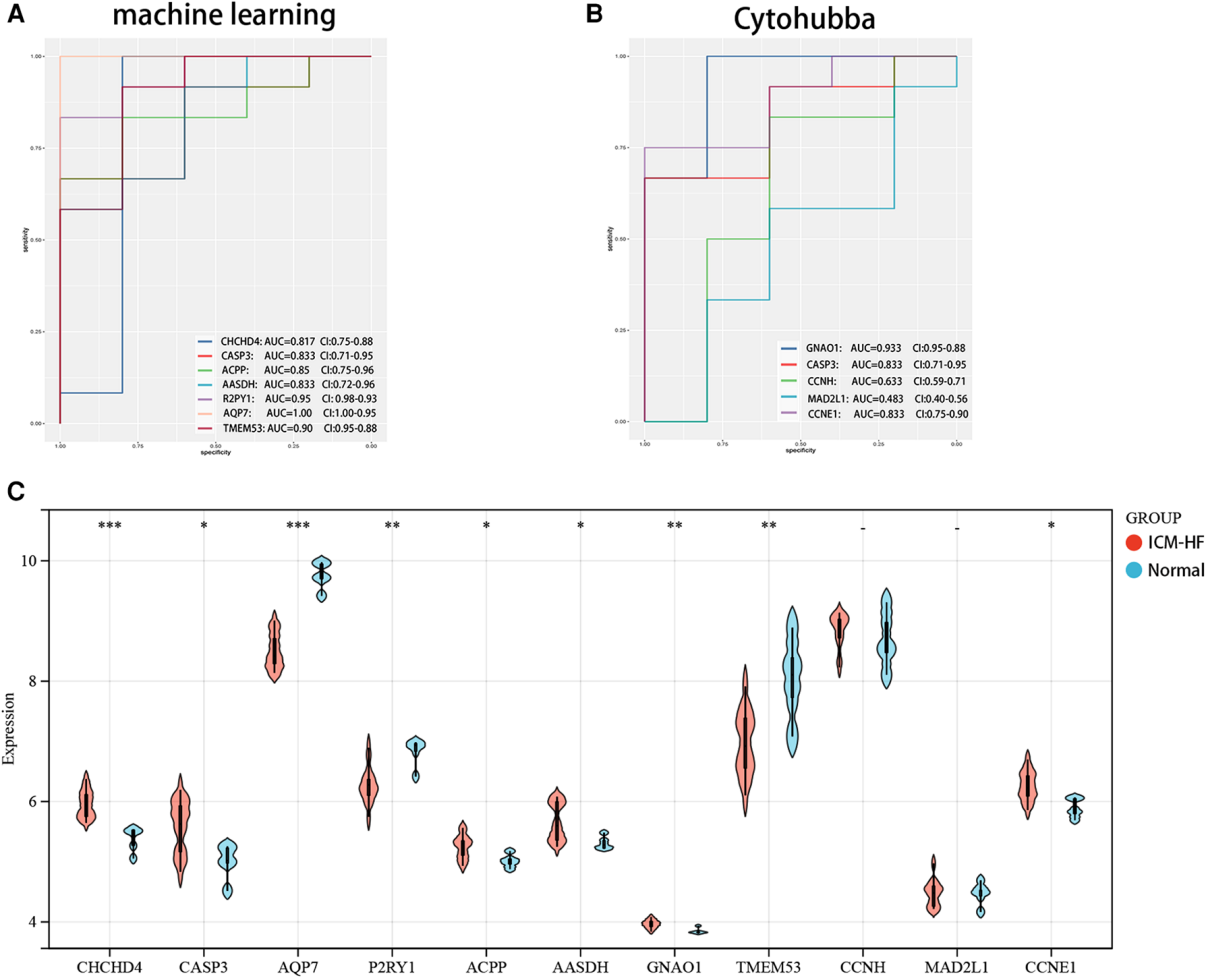
Verification of expression and diagnostic efficacy for optimal feature genes using external validation dataset. (**A**) Validation of ROC of genes screened by a machine learning algorithm. (**B**) ROC validation of genes related to the cytohubba algorithm. (**C**) Violin plot of the expression of the relevant genes in the experimental set GSE42955.

In addition, for accurate and reliable results, we further validated the expression levels of the optimal feature genes in external validation dataset GSE42955 (Figure 9). In the dataset GSE42955, the genes CCNH and MAD2L1 were not statistically significant calculated by cytohubba method. Interestingly, the 7 (CHCHD4, CASP3, ACPP, AASDH, AQP7, P2RY1, TMEM53) genes screened by ML methods all share the same trend in the above-mentioned dataset, and at the same time, they are all statistically significant.

### 3.6. GSEA analysis and ssGSEA analysis

**Figure 10:**
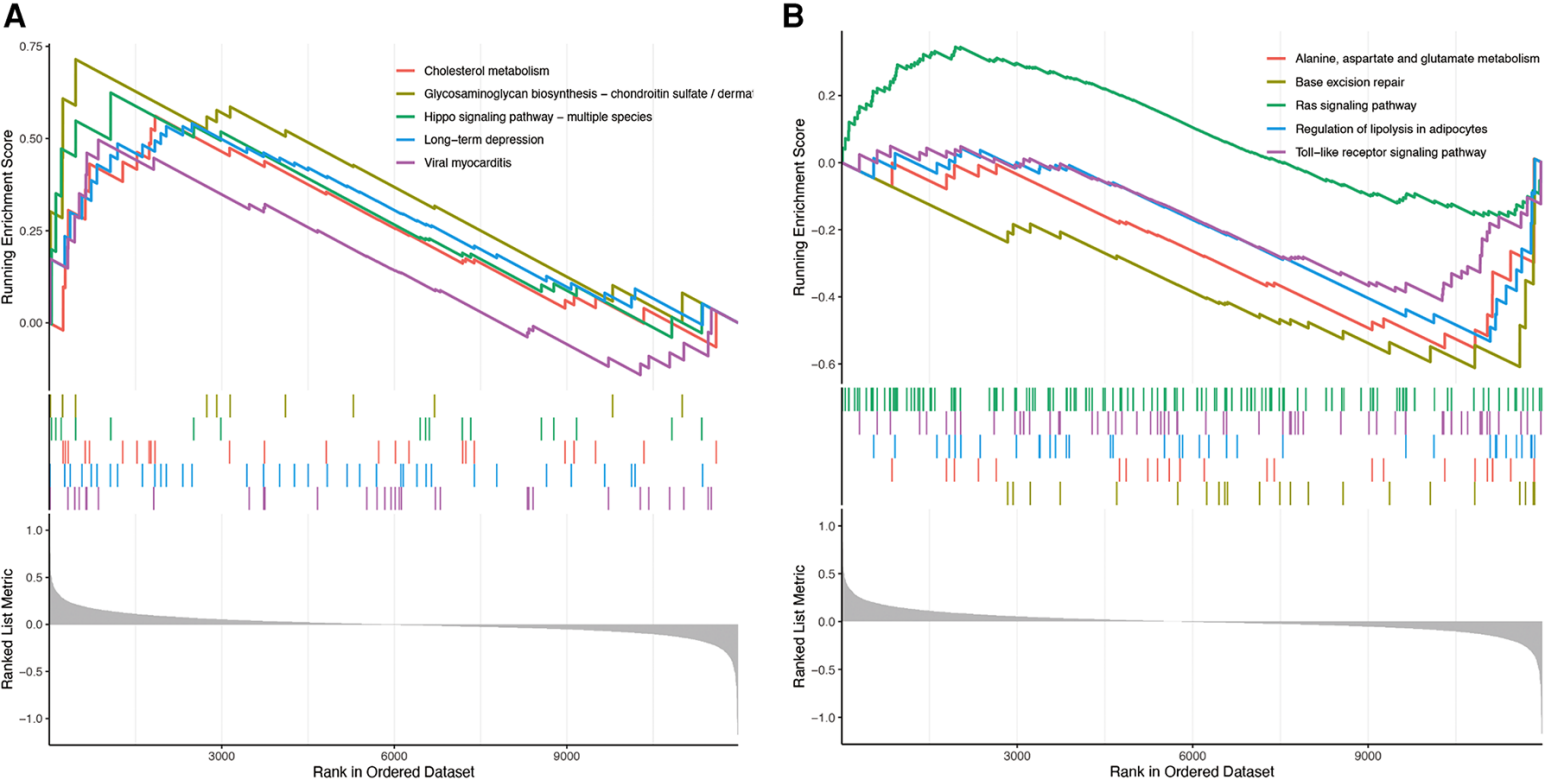
Enrichment of GSEA pathway. (**A**) GSE57345 upregulated gene pathway enriched in GSEA. (**B**) GSE57345 enriched in GSEA for downregulated gene pathway.

**Figure 11:**
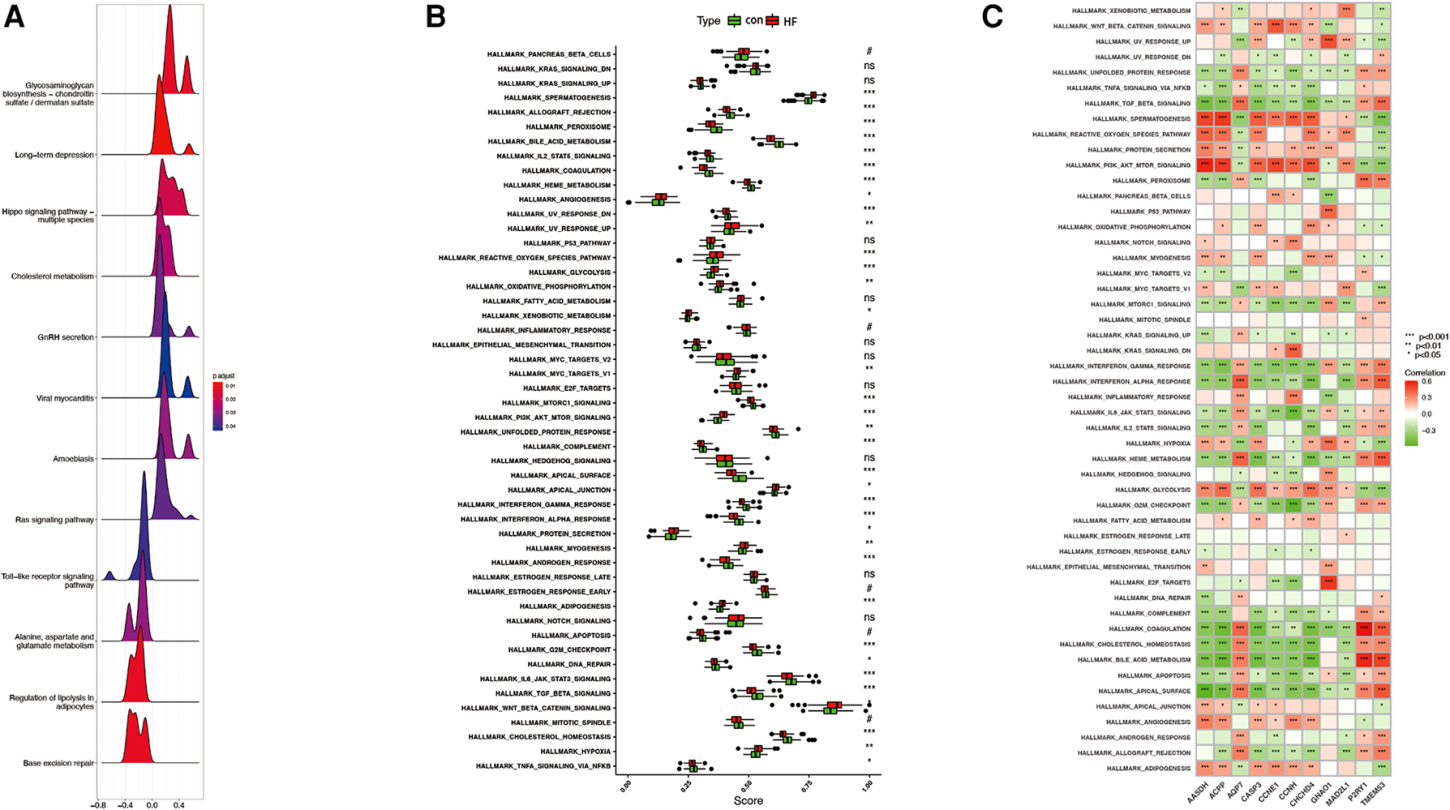
Plot of correlated differences in GSEA enrichment. (**A**) Mountain range plot of the correlation pathway of GSE57345 enrichment in GSEA. (**B**) The specific distribution of the hallmark gene sets in ICM-HF and normal samples. (**C**) Correlation analysis of the hallmark gene sets with seven optimal feature genes. Statistic tests: Wilcoxon rank-sum test (*p* < 0.2; *p < 0.05; **p < 0.01; ***p < 0.001; ns, no significance).#.

**Figure 12:**
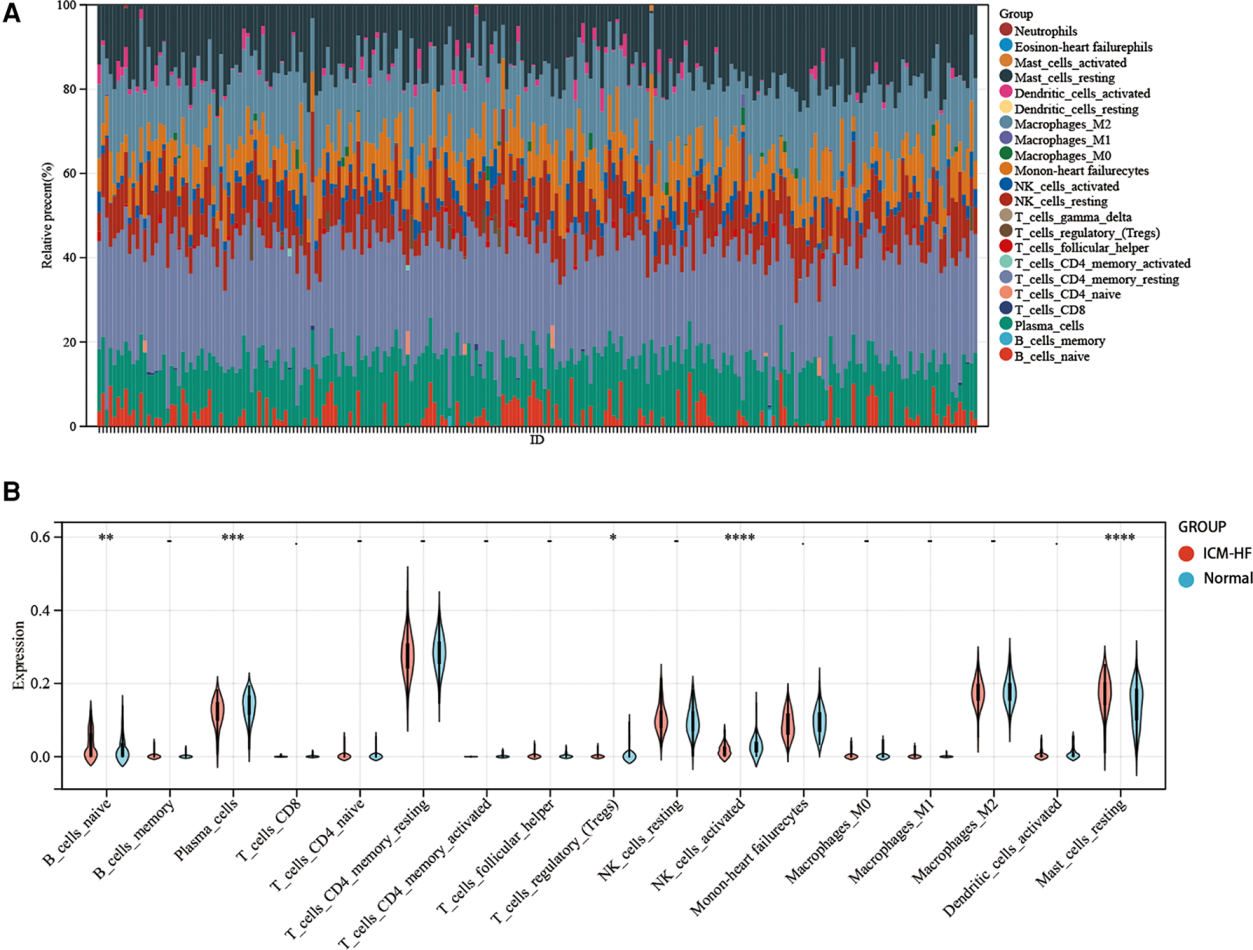
Immune infiltration analysis. (**A**) Visualization of stacked plots of various infiltrating immune cells in GSE57345. (**B**) Visualization of violin plots of infiltrating immune cells in the ICM-HF and normal groups. *p < 0.05, **p < 0.01, ***p < 0.001. Wilcoxon rank-sum test.

GSEA analysis showed that the enrichment pathway was mainly positively correlated with pathways such as cholesterol metabolism and negatively correlated with pathways such as lipolysis presentation in adipocytes (Figures 10A,B). The upregulated pathways in ssGSEA are shown in Figure 11A by mountain range plots. The relative significance of each pathway is shown by a box line plot (Figure 11B). The most significant differential pathways are mainly focused on lipid metabolism, organelle damage, and oxidative stress-related pathways. We analyzed the correlations of individual genes in the relevant pathways. The correlations of genes screened by ML and genes screened by cytohubba with the pathways are visualized in Figure 12C, with red representing positive correlations and green representing negative correlations.

### 3.7. Immune infiltration analysis

Since we observed enrichment of ICM-HF-related genes in immune regulation, immune cell infiltration analysis was performed to better elucidate the immune regulation of ICM-HF.

**Figure 13:**
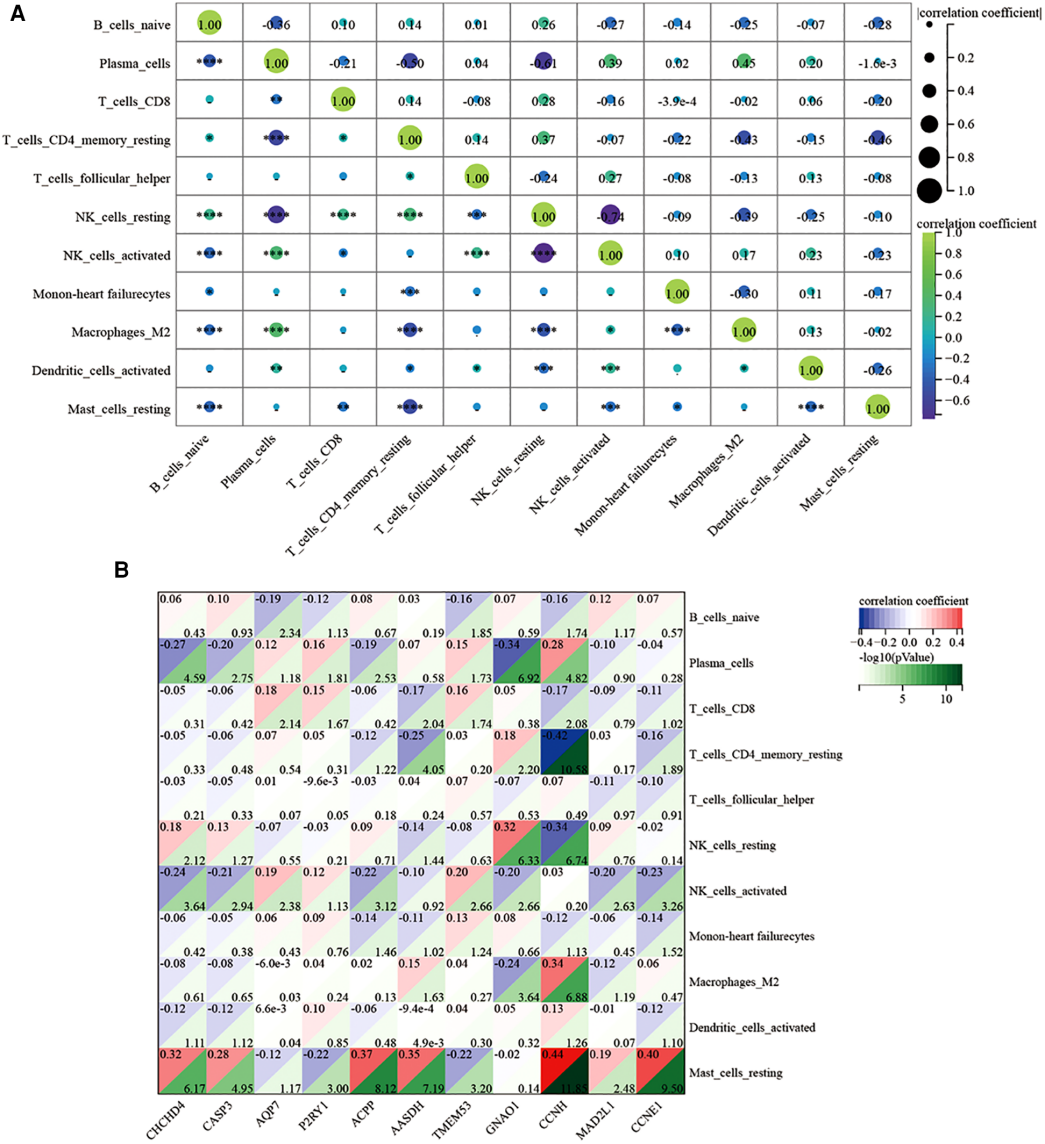
Correlation analysis of immune-related infiltration analysis. (**A**) The relative proportions of 22 immune cells types between normal samples and ICM-HF samples. in GSE57345. (**B**) Correlation analysis of 11 related disease genes and associated immune cells. *p < 0.05, **p < 0.01, ***p < 0.001.Wilcoxon rank-sum test.

Regarding the infiltration of 22 immune cells in the ICM-HF and the normal group controls shown in Figure 12A, the violin plot indicates that patients with ICM-HF have higher levels of plasma cells, naive B cells, and resting mast cells and lower levels of activated NK cells and regulatory T cells (Figure 12B). The correlation of individual immune cells is shown in Figure 13A. In general, multiple immune cells are differentially expressed in patients with ICM-HF, which can be used as potential therapeutic targets. Correlation analysis of genes and immune cells (Figure 13B) revealed positive correlations between resting mast cells and several related genes, so we hypothesize that resting mast cells may play an important role in HF due to ICM-HF and CCNH genes correlate with several immune cells, such as negative correlations with memory T cells and resting NK cells and positive correlations with plasma cells and M2 type macrophages. The CCNH gene is associated with several immune cells, such as memory T cells and resting NK cells and with plasma cells and M2 macrophages.

## 4. Discussion

The strong bioinformatics analysis community and technology repositories that have driven advances in modern genetics. Recently, an increasing number of examples of ML-driven analysis are emerging in the field of cardiovascular genetics, including coronary calcium studies (25), pulmonary hypertension (26), and multiple clinically relevant variant assays from next-generation sequencing or proteomic data (27). Modern medical practice is awash with many types of data. In cardiovascular medicine, the range and quality of diagnostic tests, such as non-invasive imaging, such as computed tomography (CT) angiography, physiological tests, and other fractional flow reserves or biomarkers, have increased over the past few decades (28). These tests provide physicians with additional complementary information upon which to base diagnostic and therapeutic decisions, which are widely accessible, less expensive and low-risk. Overall, the high prevalence of cardiovascular disease generates a large amount of patient data related to cardiovascular disease. This provides a large amount of data for training ML models and gives ML the opportunity to assist in more clinical work.

In our research, we used multiple independent algorithms. And then we identified the important genes from dataset. These ML methods are used to find valuable information from complex and large gene expression data. This has enabled researchers to explore potential influences in disease from different perspectives and using different methods. For example, it can identify genes that have not been studied in previous research and provide researchers with new insights and ideas in the study of specific diseases. We screened 7 disease-associated genes using ML (CHCHD4, CASP3, TMEM53, ACPP, AASDH, P2RY1 and AQP7). We used cytohubba to screen for 5 genes (GNAO1, CCNH, MAD2L1, CASP3, CCNE1). Among them, CASP3 was the common gene derived from cytohubba analysis and ML analysis. Based on the results of our study, overall, the diagnostic efficacy of the genes screened by ML may be better than that of cytohubba.

CASP3 is a frequently activated death protease that catalyzes the specific cleavage of many key cellular proteins and is involved in apoptotic cell death (29). Studies have demonstrated that CASP3 is involved in the inflammatory activation and immune cell aggregation in cardiovascular disease through the regulation of the Rho-kinase axis by vascular smooth muscle cells (30, 31).

According to our results, CHCHD4 expression was significantly higher in the ICM-HF group compared to normal group, so it is speculated that elevated CHCHD4 may play an important role in the ICM-HF. CHCHD4 plays a key role in oxidative protein folding in the mitochondrial membrane gap, representing a minimal thioredoxin-independent oxidoreductase, which ensures its catalytic function in mitochondrial oxidative folding (32). CHCHD4-based protein import mechanisms are essential for the maintenance of normal mitochondrial functions (33). Damaged mitochondria produce less adenosine triphosphate (ATP) and generate dangerous amounts of ROS. Accumulated ROS may damage mitochondrial DNA, cell membranes, and respiratory complex proteins, leading to catastrophic oxidative damage and cell death (34). We hypothesize that the elevated CHCHD4 protein in the ICM-HF group is a compensatory manifestation of mitochondrial damage due to hypoxia in cardiomyocytes caused by myocardial ischemia. It is expected to be a new target for the diagnosis and treatment of ICM-HF.

Studies have shown that Aquaporins (AQPs) are involved in the regulation of cardiovascular function and the development of related diseases, particularly in cerebral ischemia, congestive HF, hypertension, and angiogenesis (35). AQP7 in AQPs is a hydroglycerol channel protein that is mainly distributed in proximal renal tubules, cardiac muscles, and adipose tissue. Studies have shown that the heart is the second most expressed tissue after adipose tissue for AQP7 mRNA (36). However, the role of AQP7 in the myocardium has been barely investigated. In the above study, AQP7 expression was significantly lower in the disease group than in the control group. During periods of high energy demand and metabolic stress, lipolysis increases and converts triglycerides to free fatty acids and glycerol. AQP7 controls glycerol efflux under these conditions, and the exported glycerol is then taken up by other cells and used as a backbone for energy requirements during high energy demands (37). ICM-HF is often accompanied by the loss of energy metabolic function and disturbances in lipid metabolism. AQP7 is required for carbohydrate metabolism, complex lipid biosynthesis, urea/arginine metabolism, redox homeostasis, amino acid metabolism, and nucleotide metabolism. Thus, AQP7 plays a critical role in regulating lipid metabolism (38).

The analysis with GO, KEGG, and GSEA pathways was enriched in cholesterol metabolism, regulation of lipolysis in adipocytes, and amino acid metabolism. The presence of a significant downregulation of AQP7 in the ICM-HF group relative to the control group was confirmed in our experimental group and validation group. AQP7's deficiency appears to impair metabolic adaptation during cardiac overload by limiting glycerol uptake and reducing intracellular ATP levels (39). This is of fundamental importance because cardiomyocyte metabolism is dependent on fatty acids, but they are converted to glucose and glycerol as energy substrates when the heart is overloaded (40). Overall, AQP7's deficiency may exacerbate the damage to the energy metabolism of the ischemic myocardium, ultimately leading to HF.

P2RY1 is a G protein-coupled receptor in which ADP is a physiological agonist that actively couples to phospholipase C *via* Gαq, thereby triggering the release of intracellular stores of Ca^2+^ (41). HF is characterized by the reduced contractile function of cardiac myocytes, resulting in reduced systolic left ventricular contraction. Defective myocardial contractility is associated with impaired excitation–contraction (EC) coupling, a mechanism that converts electrical stimulation from pacemaker cells into contraction through the release of large amounts of Ca^2+^ from the sarcoplasmic reticulum (SR) (42). Interestingly, studies have shown that the dysregulation of intracellular calcium homeostasis in cardiac myocytes is an important factor in exacerbating the cardiovascular disease (43). We found a significant downregulation of P2RY1 in ICM-HF group. Therefore, we consider that P2RY1 may play an important role in ICM-HF.

Growing evidence suggests that immune cell infiltration of the myocardium has a detrimental effect on cardiac function (44–46). Immune cell profiles differ significantly in healthy and diseased hearts (46). In this study, we found that naive B cells and resting mast cells were significantly elevated in the disease genome. Plasma cells, regulatory T cells, and activated NK cells were significantly downregulated within the ICM-HF group. Mast cells exacerbate the progression of ischemic HF by activating matrix metalloproteinases and cardiac fibrosis (47). Myocardial fibrosis and resistance to neo-angiogenesis caused by dysfunction of regulatory T cells are, on the other hand, a very critical step in the pathological progression of cardiovascular disease (48, 49). Through our analysis, we consider that the infiltration of immune cells may provide new ideas and insights in the diagnosis and treatment of ICM-HF.

We identified 11 potentially significant pathogenic genes in ICM-HF through ML and PPI analysis. We focused on the possible roles of the mitochondrial damage and apoptosis genes CHCHD4 and CASP3 and the lipid metabolism regulatory genes AQP7 and P2RY1 in disease development. The differential expression of TMEM53, ACPP and AASDH genes may also play an important role in ICM-HF, but these genes have been barely investigated, so little is known about the function of these genes. These genes may play a critical role in the development and progression of the ICM-HF, and their functions and mechanisms need to be further explored. This is a reflection of the innovation and excellence of ML in the medical field, which has been able to identify many influences that have not been identified by previous studies. At the same time, it can also provide new directions and ideas for the study of ICM-HF. Overall, our study demonstrates for the first time the promising potential of a combined WGCNA and ML approach in transcriptomic data for ICM-HF. Our findings suggest that ML modeling of genome-wide transcriptomic data from cardiac samples collected by clinical heart biopsy can explore potential biomarkers. Finally, our study prioritizes previously unknown genes and genes that have not been studied in ICM-HF as potential candidate biomarkers. Our work can serve as an important part of future research in the field of ICM-HF. Interestingly, we found that after validation by external gene set GSE42955, all seven genes screened by ML were well validated, while CCNH and MAD2L1 of the five genes screened by PPI were not well validated. This also reflects the superiority of ML compared to common bioinformatics algorithms.

Some limitations of the present study should be noted. First, this is a retrospective study, and further prospective experiments need to be designed. Second, this study can further design animal experiments to explore the mechanism of validating related genes in ICM-HF. Third, the accuracy of our chosen SVM algorithm is only 0.758. However, the use of multiple ML algorithms to analyze the potential causative genes depicting ICM-HF increases the credibility of the study to some extent and explores the correlation between the status of immune infiltrating cells in the tissues of ischemic HF and causative genes, which are biomarkers that may provide guidance for the diagnosis and treatment of patients with ICM-HF.

## Data Availability

The original contributions presented in the study are included in the article/**Supplementary Material**, further inquiries can be directed to the corresponding author.
