# Supplementary material for: WGCNA combined with machine learning algorithms for analyzing key genes and immune cell infiltration in heart failure due to ischemic cardiomyopathy
Source: Front Cardiovasc Med. 2023 Mar 17;10:1058834. doi: 10.3389/fcvm.2023.1058834 (PMC10064046; doi:10.3389/fcvm.2023.1058834)
Supplement: Supplementary file 1 [file Datasheet1.pdf]

Supplementary materials

WGCNA combined with machine learning algorithms for analyzing key genes and immune cell infiltration in heart failure due to ischemic cardiomyopathy

XiangJin Kong[1,2†], HouRong Sun[1,2†], KaiMing Wei[1,2], LingWei Meng[1,2], Xin Lv [1,2], ChuanZhen Liu[1,2], FuShun Lin[1,2], XingHua Gu[1,2*]

[1]Qilu Hospital, Cheeloo College of Medicine, Shandong University
[2]Department of Cardiovascular Surgery, Qilu Hospital of Shandong University, Jinan Shandong, China

* Correspondence:
XingHua Gu*
guxh2005@126.com

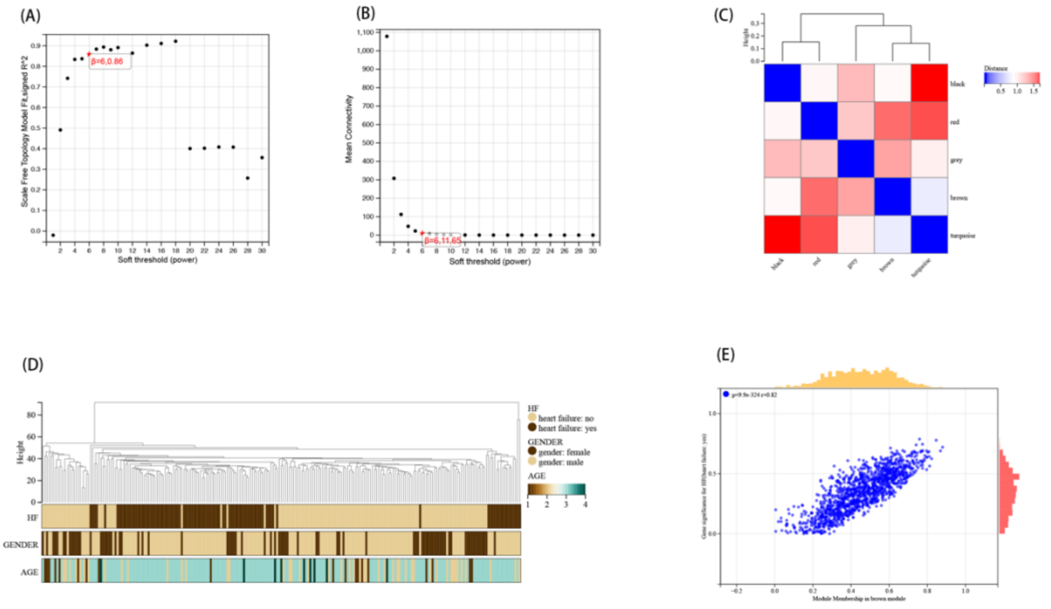

Figure S1 (A, B) Network topology for analyzing various soft thresholds. (E) The heatmap of topological overlap in gene networks. (C) The heatmap of topological overlap in gene networks. (D) Gene and trait clustering dendrograms. Gene clustering trees (dendrograms) obtained by hierarchical clustering of neighbor-based differences. (E) Scatter plot of the featured gene modules,

showing brown modules with good correlations.

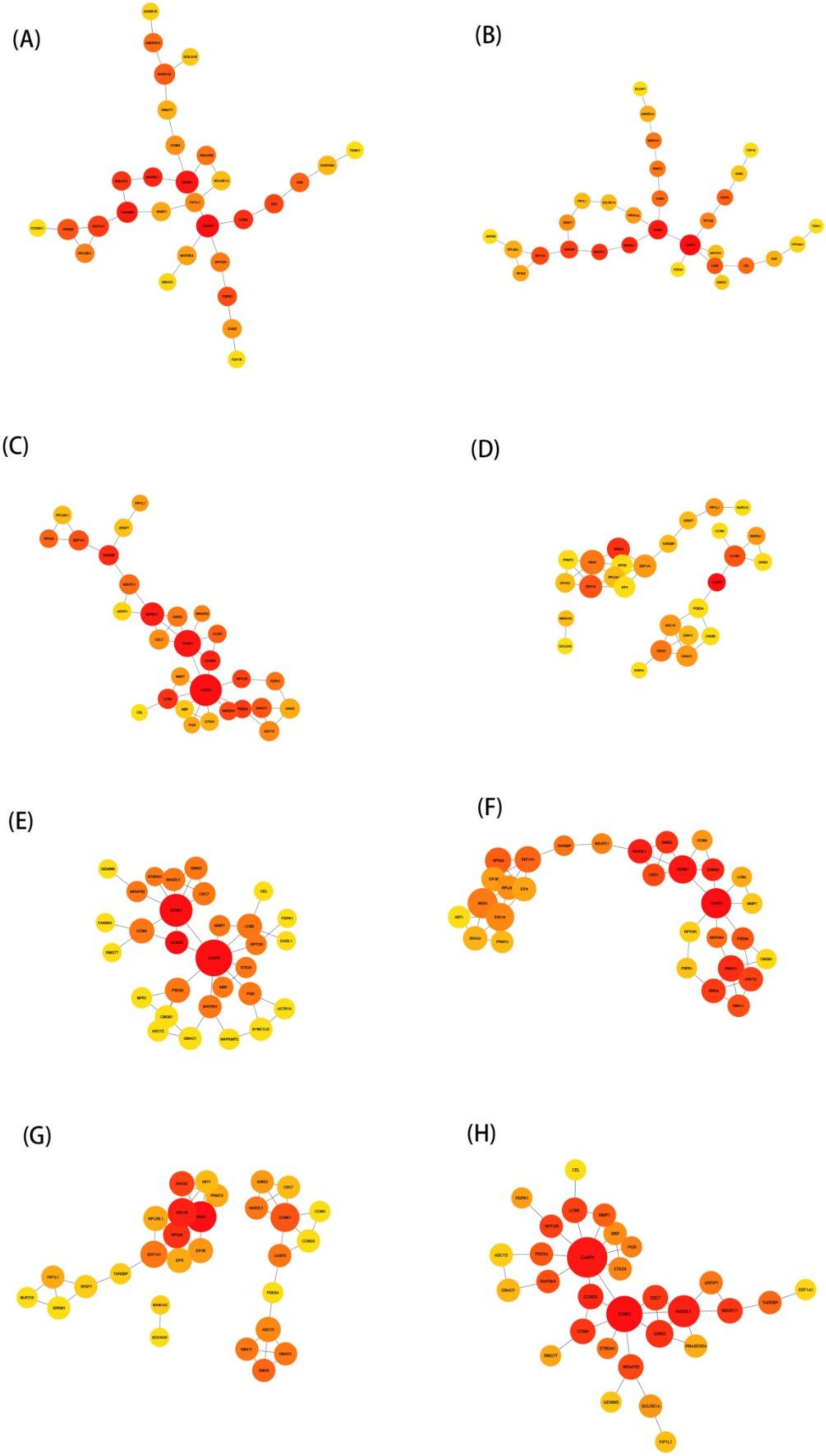
(A)
(B)
(C)
(D)
(E)
(F)
(G)
(H)

Figure S2 The top 30 significant genes calculated using eight different cytoHubba algorithms. (A) Betweenness (B) BottleNeck (C) Closeness (D) Degree (E) EcCentricity (F) EPC (G) Radiality (H) MCC.

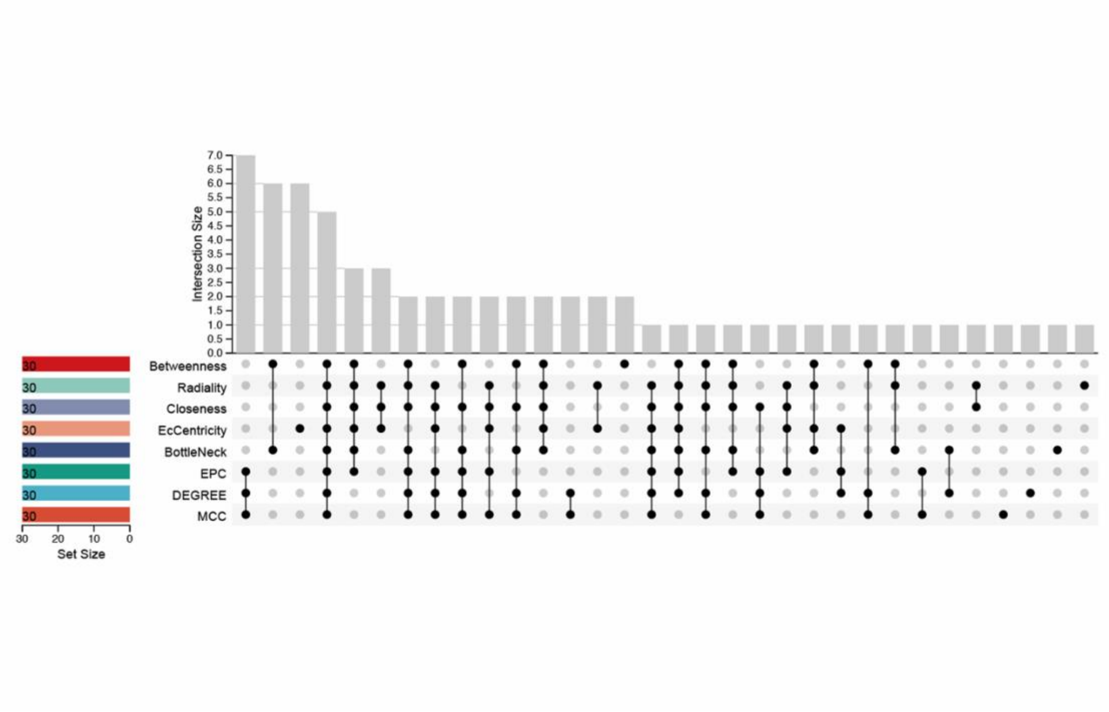

Figure S3: UpSet plot showing the intersection genogram between eight different cytohubba algorithms.
