# Supplementary figures and images for: WGCNA combined with machine learning algorithms for analyzing key genes and immune cell infiltration in heart failure due to ischemic cardiomyopathy
Source: Front Cardiovasc Med. 2023 Mar 17;10:1058834. doi: 10.3389/fcvm.2023.1058834 (PMC10064046; doi:10.3389/fcvm.2023.1058834)

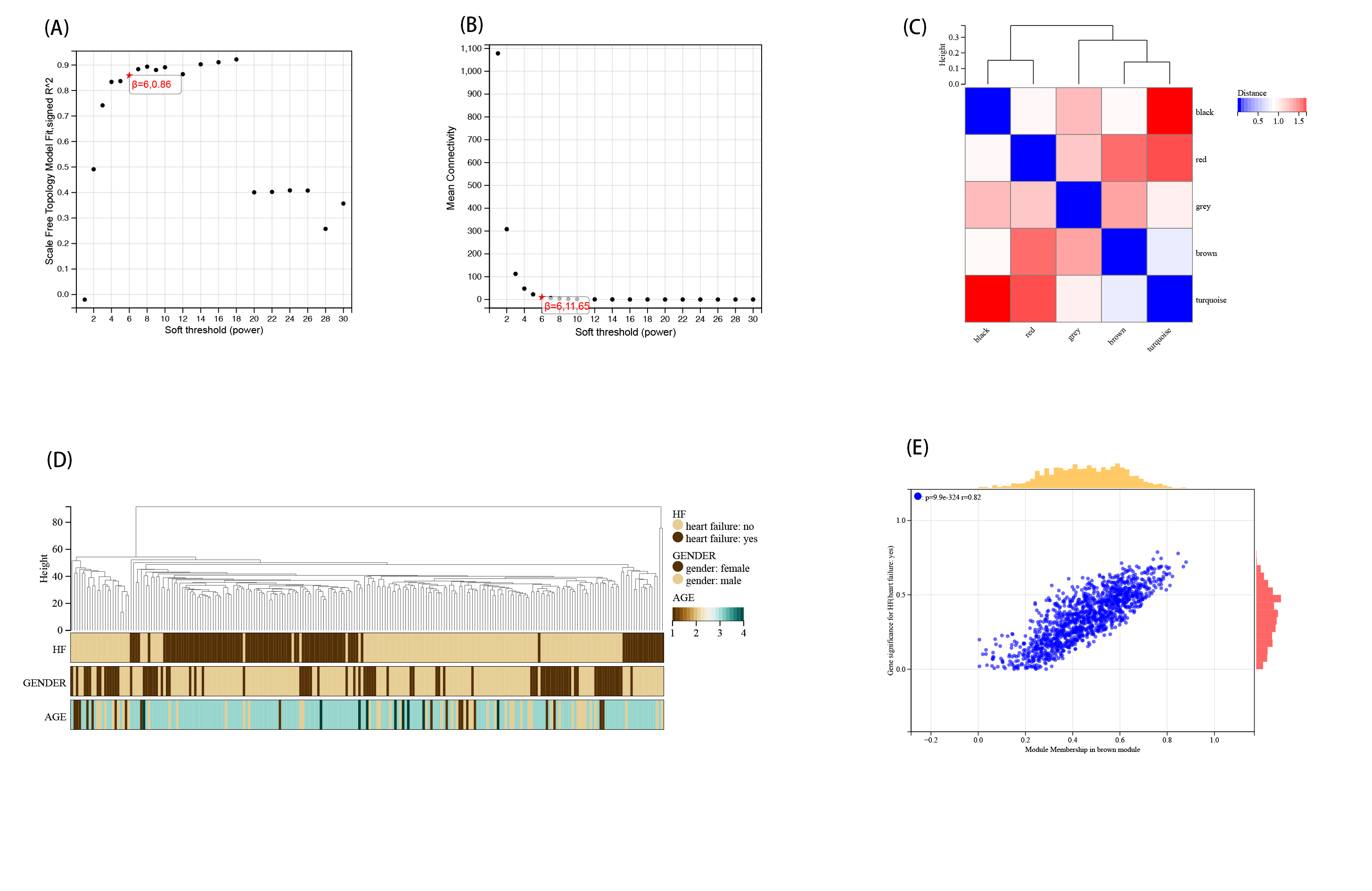

Supplement: Supplementary file 3 [file Image1.tif]

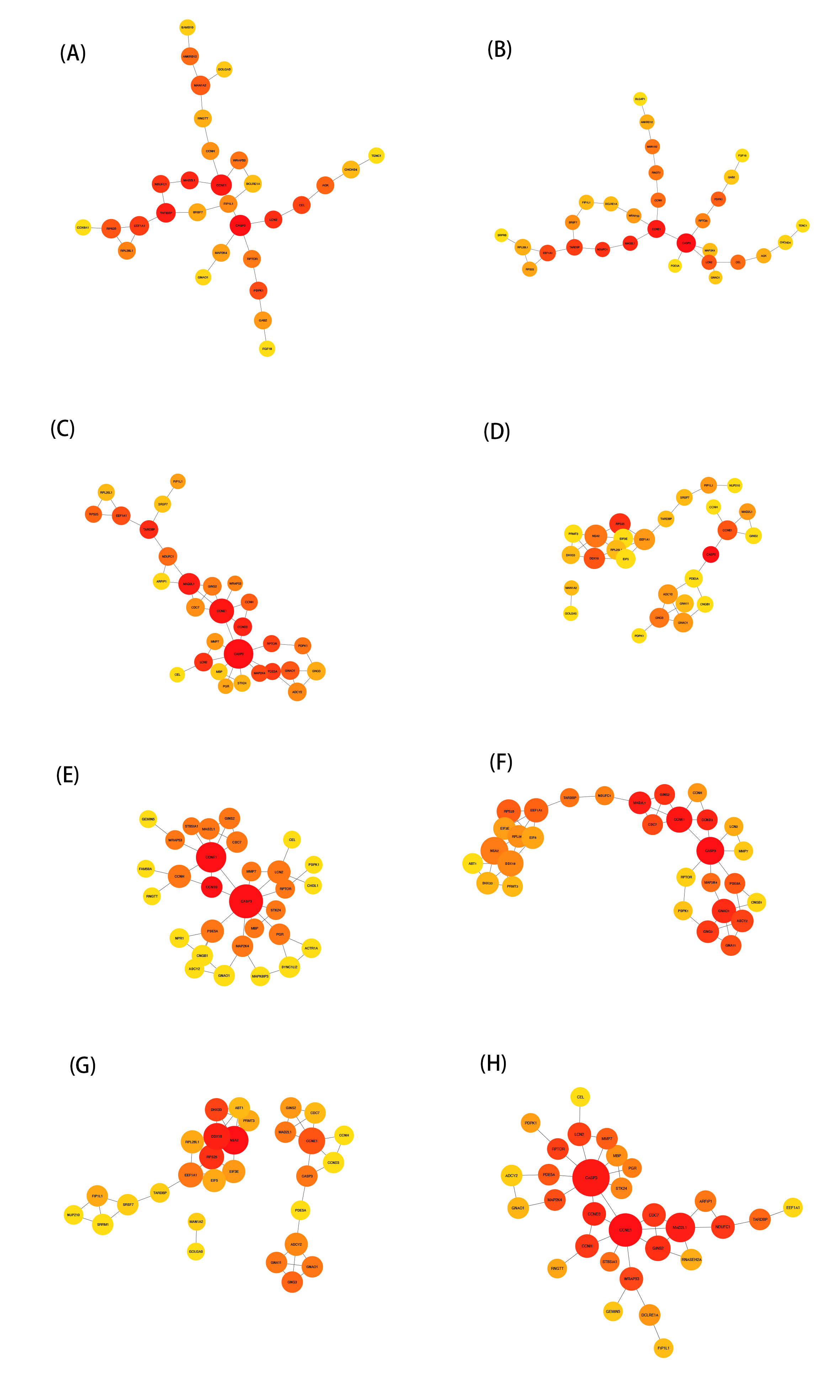

Supplement: Supplementary file 4 [file Image2.tif]

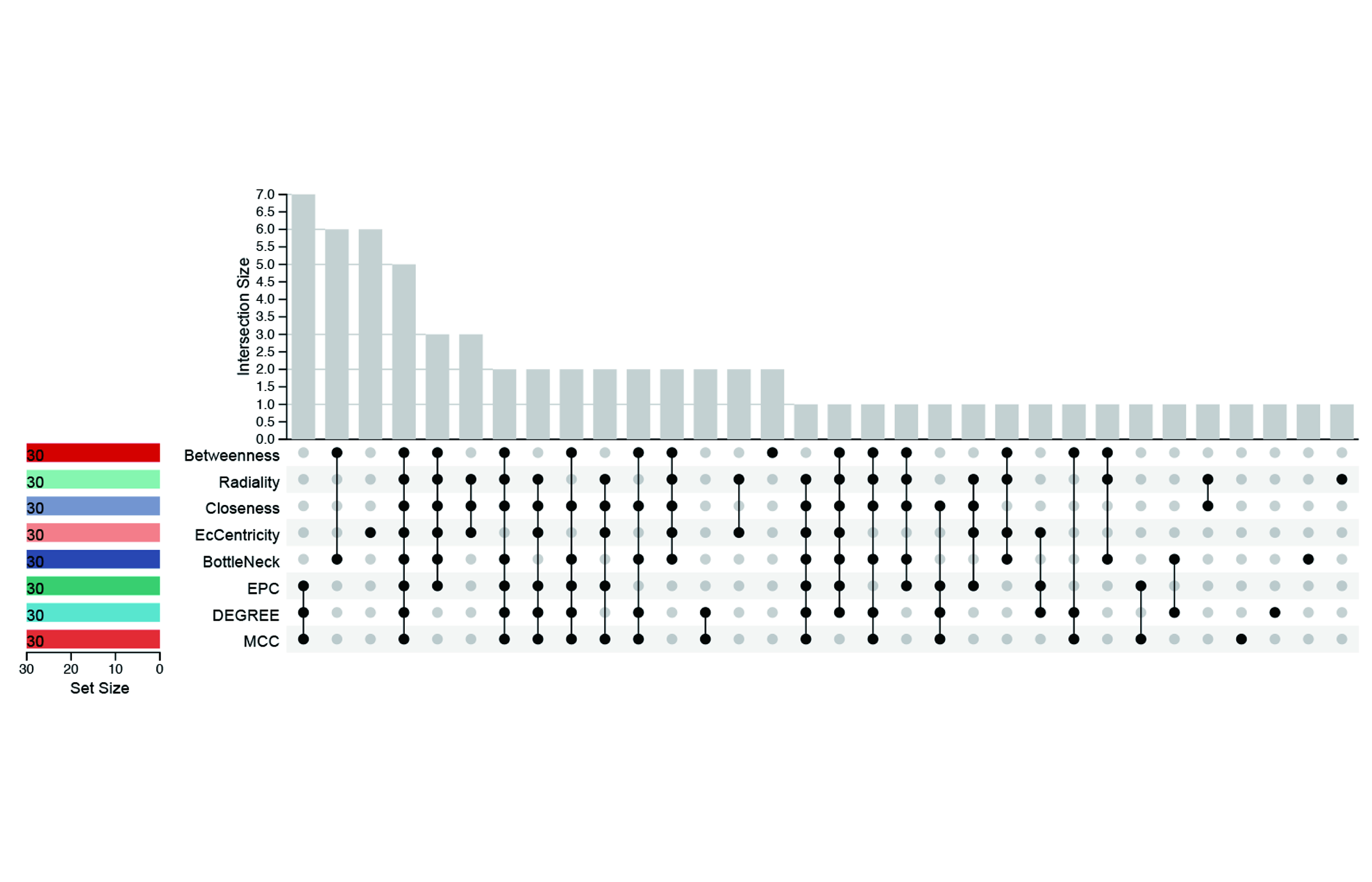

Supplement: Supplementary file 5 [file Image3.tif]
